# *N*-(2-Thienylmethyl­ene)naphthalen-1-amine

**DOI:** 10.1107/S1600536809041774

**Published:** 2009-10-17

**Authors:** Xuquan Tao, Hui Cui

**Affiliations:** a College of Materials Science and Engineering, Liaocheng University, Shandong 252059, People’s Republic of China; b College of Chemistry and Chemical Engineering, Liaocheng University, Shandong 252059, People’s Republic of China

## Abstract

In the title compound, C_15_H_11_NS, the dihedral angle between the thio­phene and 1-naphthyl rings is 31.42 (11)°. The mol­ecule adopts a *trans* configuration about the central C=N bond. In the crystal, the mol­ecules are connected *via* weak C—H⋯π inter­actions.

## Related literature

The condensation of primary amines with carbonyl compounds yields Schiff bases, see: Dey *et al.* (1981 ▶). For the chemistry and applications of Schiff bases, see: Doine (1985 ▶); Opstal *et al.* (2002 ▶).
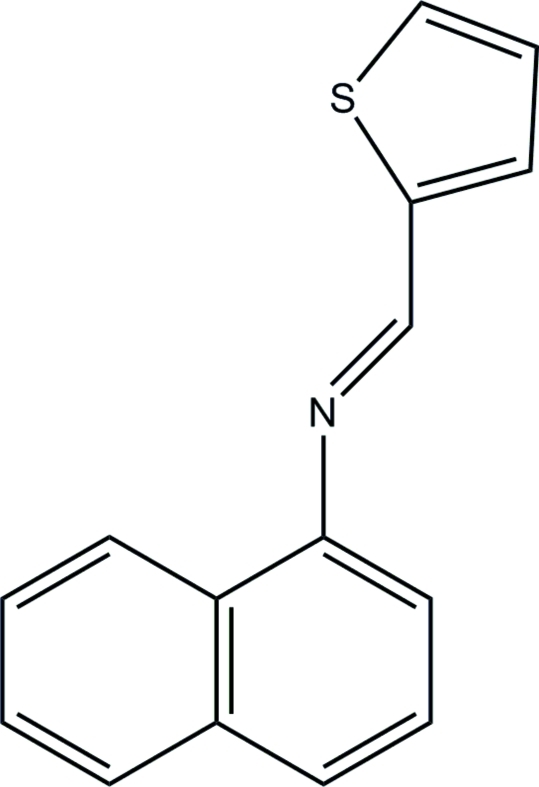


## Experimental

### 

#### Crystal data

C_15_H_11_NS
*M* *_r_* = 237.31
Orthorhombic, 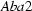

*a* = 10.7793 (12) Å
*b* = 21.260 (2) Å
*c* = 10.7244 (10) Å
*V* = 2457.7 (4) Å^3^
*Z* = 8
Mo *K*α radiation
μ = 0.24 mm^−1^
*T* = 298 K
0.40 × 0.38 × 0.18 mm

#### Data collection

Bruker SMART APEX CCD area-detector diffractometer
Absorption correction: multi-scan (*SADABS*; Sheldrick, 1996 ▶) *T*_min_ = 0.911, *T*_max_ = 0.958
5898 measured reflections
2115 independent reflections
1613 reflections with *I* > 2σ(*I*)
*R*_int_ = 0.038

#### Refinement

*R*[*F* ^2^ > 2σ(*F* ^2^)] = 0.036
*wR*(*F* ^2^) = 0.076
*S* = 1.05
2115 reflections
154 parameters
1 restraint
H-atom parameters constrained
Δρ_max_ = 0.14 e Å^−3^
Δρ_min_ = −0.22 e Å^−3^
Absolute structure: Flack (1983 ▶), 965 Friedel pairs
Flack parameter: 0.01 (9)

### 

Data collection: *SMART* (Siemens, 1996 ▶); cell refinement: *SAINT* (Siemens, 1996 ▶); data reduction: *SAINT*; program(s) used to solve structure: *SHELXS97* (Sheldrick, 2008 ▶); program(s) used to refine structure: *SHELXL97* (Sheldrick, 2008 ▶); molecular graphics: *SHELXTL* (Sheldrick, 2008 ▶); software used to prepare material for publication: *SHELXTL*.

## Supplementary Material

Crystal structure: contains datablocks I, global. DOI: 10.1107/S1600536809041774/bx2244sup1.cif

Structure factors: contains datablocks I. DOI: 10.1107/S1600536809041774/bx2244Isup2.hkl

Additional supplementary materials:  crystallographic information; 3D view; checkCIF report

## Figures and Tables

**Table 1:** Hydrogen-bond geometry (Å, °)

*D*—H⋯*A*	*D*—H	H⋯*A*	*D*⋯*A*	*D*—H⋯*A*
C13—H13⋯*Cg*1^i^	0.93	2.87	3.783 (3)	168

Symmetry code: (i) 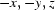. *Cg*1 is the centroid of the S1,C2–C5 ring.

**Fig. 1.**
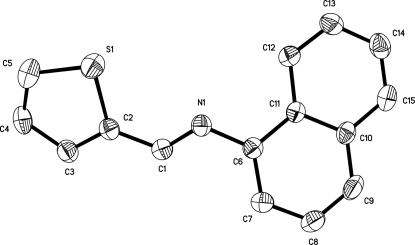
The content of asymmetric unit of the title compound showing the atomic numbering scheme and 30% probability displacement ellipsoids.

**Fig. 2.**
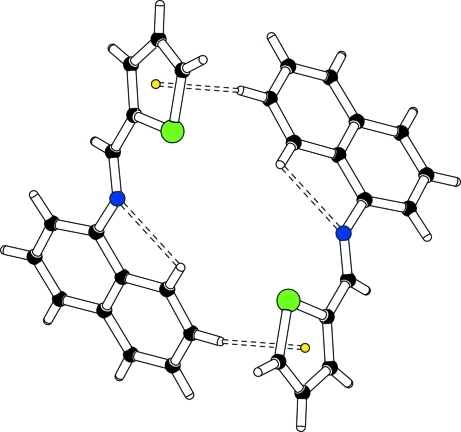
Part of the crystal structure of (I) showing the hydrogen bond and C-H···π interactions.

## supplementary crystallographic information

### Comment

The condensation of primary amines with carbonyl compounds yields Schiff bases (Dey *et al.*, 1981). In the recent years, there has been considerable interest in the chemistry of Schiff bases (Doine, 1985). This is due to the fact that Schiff bases offer opportunities for inducing substrate chirality, tuning the metal centred electronic factor, enhancing the solubility and stability of either homogeneous or heterogeneous catalysts (Opstal *et al.*, 2002). We report here the synthesis and crystal structure of , (I) present a new compound, 2-(2-(naphthalen-1-yl)vinyl)thiophene schiff base, (I) in this paper.

The structure of (I) consists of 1- naphthyl ring covalently linked to a thiophene ring by an azomethine bond with more stable E isomer being observed. The mean plane of the 1-naphthyl ring is twisted by 35.4 (2)° from the azomethine bond to which is connected. The molecule adopts a trans configuration about the central C═N bond. In the crystal structure the molecules are interconnected via a C—H···π interactions, and the molecular structure is stabilized by one intramolecular C—H···N hydrogen bond, Table 1, Fig 2.

### Experimental

Naphthylamine(10 mmol), thiophene-2-carbaldehyde (20 mmol) and 20 ml ethanol were mixed in 50 ml flask. After stirring 3 h at 303 K, the resulting mixture was recrystalized from ethanol, affording the title compound as a red crystalline solid. The single crystals were obtained methylene dichloride and n-hexane solution. The Elemental analysis: calculated for C_15_H_11_NS: C 75.91, H 4.67, N 5.90%; found: C 75.82, H 4.54, N 9.57%.

### Refinement

All H atoms were placed in geometrically idealized positions (C—H distances is 0.93 Å) and treated as riding on their parent atoms, with *U*_iso_(H) = 1.2 *U*_eq_(C).

### Crystal data

**Table:**

C_15_H_11_NS	*D*_x_ = 1.283 Mg m^−^^3^
*M**_r_* = 237.31	Mo *K*α radiation, λ = 0.71073 Å
Orthorhombic, *A**b**a*2	Cell parameters from 2185 reflections
*a* = 10.7793 (12) Å	θ = 2.7–22.1°
*b* = 21.260 (2) Å	µ = 0.24 mm^−^^1^
*c* = 10.7244 (10) Å	*T* = 298 K
*V* = 2457.7 (4) Å^3^	Block, red
*Z* = 8	0.40 × 0.38 × 0.18 mm
*F*(000) = 992	

### Data collection

**Table:**

Bruker SMART APEX CCD area-detector diffractometer	2115 independent reflections
Radiation source: fine-focus sealed tube	1613 reflections with *I* > 2σ(*I*)
graphite	*R*_int_ = 0.038
phi and ω scans	θ_max_ = 25.0°, θ_min_ = 1.9°
Absorption correction: multi-scan (*SADABS*; Sheldrick, 1996)	*h* = −12→12
*T*_min_ = 0.911, *T*_max_ = 0.958	*k* = −25→15
5898 measured reflections	*l* = −11→12

### Refinement

**Table:**

Refinement on *F*^2^	Secondary atom site location: difference Fourier map
Least-squares matrix: full	Hydrogen site location: inferred from neighbouring sites
*R*[*F*^2^ > 2σ(*F*^2^)] = 0.036	H-atom parameters constrained
*wR*(*F*^2^) = 0.076	*w* = 1/[σ^2^(*F*_o_^2^) + (0.0332*P*)^2^] where *P* = (*F*_o_^2^ + 2*F*_c_^2^)/3
*S* = 1.05	(Δ/σ)_max_ < 0.001
2115 reflections	Δρ_max_ = 0.14 e Å^−^^3^
154 parameters	Δρ_min_ = −0.22 e Å^−^^3^
1 restraint	Absolute structure: Flack (1983), 965 Friedel pairs
Primary atom site location: structure-invariant direct methods	Flack parameter: 0.01 (9)

### Special details

**Table:**

Geometry. All e.s.d.'s (except the e.s.d. in the dihedral angle between two l.s. planes) are estimated using the full covariance matrix. The cell e.s.d.'s are taken into account individually in the estimation of e.s.d.'s in distances, angles and torsion angles; correlations between e.s.d.'s in cell parameters are only used when they are defined by crystal symmetry. An approximate (isotropic) treatment of cell e.s.d.'s is used for estimating e.s.d.'s involving l.s. planes.
Refinement. Refinement of *F*^2^ against ALL reflections. The weighted *R*-factor *wR* and goodness of fit *S* are based on *F*^2^, conventional *R*-factors *R* are based on *F*, with *F* set to zero for negative *F*^2^. The threshold expression of *F*^2^ > σ(*F*^2^) is used only for calculating *R*-factors(gt) *etc*. and is not relevant to the choice of reflections for refinement. *R*-factors based on *F*^2^ are statistically about twice as large as those based on *F*, and *R*- factors based on ALL data will be even larger.

### Fractional atomic coordinates and isotropic or equivalent isotropic displacement parameters (Å^2^)

**Table:**

	*x*	*y*	*z*	*U*_iso_*/*U*_eq_	
S1	0.21178 (7)	0.07346 (3)	0.26408 (7)	0.0623 (2)	
N1	0.0437 (2)	0.14325 (8)	0.08130 (17)	0.0461 (5)	
C1	0.1600 (2)	0.14895 (11)	0.0624 (2)	0.0479 (6)	
H1	0.1865	0.1716	−0.0068	0.058*	
C2	0.2522 (2)	0.12187 (12)	0.1433 (2)	0.0473 (6)	
C3	0.3789 (2)	0.12590 (13)	0.1330 (2)	0.0590 (7)	
H3	0.4188	0.1494	0.0718	0.071*	
C4	0.4419 (3)	0.09107 (14)	0.2238 (3)	0.0657 (8)	
H4	0.5279	0.0893	0.2306	0.079*	
C5	0.3642 (3)	0.06064 (14)	0.2997 (2)	0.0684 (9)	
H5	0.3903	0.0351	0.3651	0.082*	
C6	−0.0400 (2)	0.16984 (11)	−0.0053 (2)	0.0443 (6)	
C7	−0.0183 (3)	0.22590 (13)	−0.0651 (3)	0.0589 (7)	
H7	0.0523	0.2491	−0.0462	0.071*	
C8	−0.1020 (3)	0.24811 (14)	−0.1541 (3)	0.0713 (8)	
H8	−0.0853	0.2857	−0.1952	0.086*	
C9	−0.2072 (3)	0.21592 (14)	−0.1819 (3)	0.0645 (8)	
H9	−0.2608	0.2313	−0.2426	0.077*	
C10	−0.2360 (2)	0.15979 (12)	−0.1201 (2)	0.0478 (6)	
C11	−0.1523 (2)	0.13664 (11)	−0.0279 (2)	0.0411 (6)	
C12	−0.1834 (3)	0.08006 (11)	0.0346 (3)	0.0502 (7)	
H12	−0.1291	0.0633	0.0932	0.060*	
C13	−0.2930 (3)	0.04964 (15)	0.0095 (3)	0.0608 (9)	
H13	−0.3135	0.0132	0.0529	0.073*	
C14	−0.3738 (3)	0.07315 (14)	−0.0811 (3)	0.0637 (8)	
H14	−0.4475	0.0520	−0.0978	0.076*	
C15	−0.3464 (2)	0.12602 (14)	−0.1444 (2)	0.0577 (7)	
H15	−0.4009	0.1406	−0.2051	0.069*	

### Atomic displacement parameters (Å^2^)

**Table:**

	*U*^11^	*U*^22^	*U*^33^	*U*^12^	*U*^13^	*U*^23^
S1	0.0579 (4)	0.0738 (5)	0.0552 (4)	0.0094 (4)	0.0061 (4)	0.0063 (4)
N1	0.0454 (14)	0.0480 (12)	0.0448 (11)	0.0009 (10)	−0.0020 (10)	−0.0021 (10)
C1	0.0512 (18)	0.0478 (15)	0.0447 (14)	0.0005 (13)	0.0007 (14)	−0.0021 (11)
C2	0.0463 (15)	0.0472 (14)	0.0485 (14)	−0.0001 (12)	−0.0004 (12)	−0.0075 (12)
C3	0.0502 (18)	0.0651 (17)	0.0617 (16)	−0.0062 (14)	−0.0018 (14)	0.0004 (15)
C4	0.0409 (17)	0.086 (2)	0.0697 (18)	0.0033 (15)	−0.0091 (15)	−0.0108 (17)
C5	0.068 (2)	0.081 (2)	0.0557 (19)	0.0227 (17)	−0.0098 (15)	−0.0066 (15)
C6	0.0417 (16)	0.0465 (15)	0.0445 (14)	0.0047 (13)	0.0049 (12)	−0.0015 (13)
C7	0.0542 (18)	0.0503 (17)	0.0724 (17)	−0.0043 (14)	0.0003 (14)	0.0104 (15)
C8	0.067 (2)	0.0600 (18)	0.087 (2)	0.0053 (17)	0.0011 (18)	0.0265 (17)
C9	0.0610 (19)	0.0690 (19)	0.0634 (16)	0.0200 (17)	−0.0064 (15)	0.0175 (16)
C10	0.0453 (16)	0.0531 (15)	0.0450 (13)	0.0116 (13)	0.0026 (12)	−0.0004 (13)
C11	0.0414 (15)	0.0440 (14)	0.0380 (12)	0.0053 (12)	0.0009 (11)	−0.0032 (12)
C12	0.055 (2)	0.0485 (16)	0.0471 (14)	0.0007 (14)	−0.0011 (12)	0.0038 (12)
C13	0.060 (2)	0.0553 (16)	0.0668 (18)	−0.0102 (16)	−0.0012 (15)	0.0014 (15)
C14	0.0516 (19)	0.060 (2)	0.080 (2)	−0.0047 (15)	−0.0107 (16)	−0.0131 (17)
C15	0.0547 (18)	0.0625 (19)	0.0558 (15)	0.0154 (15)	−0.0111 (13)	−0.0086 (15)

### Geometric parameters (Å, °)

**Table:**

S1—C5	1.709 (3)	C7—H7	0.9300
S1—C2	1.711 (3)	C8—C9	1.358 (4)
N1—C1	1.276 (3)	C8—H8	0.9300
N1—C6	1.413 (3)	C9—C10	1.400 (4)
C1—C2	1.440 (3)	C9—H9	0.9300
C1—H1	0.9300	C10—C15	1.414 (4)
C2—C3	1.372 (4)	C10—C11	1.427 (3)
C3—C4	1.400 (4)	C11—C12	1.417 (3)
C3—H3	0.9300	C12—C13	1.374 (4)
C4—C5	1.335 (4)	C12—H12	0.9300
C4—H4	0.9300	C13—C14	1.396 (4)
C5—H5	0.9300	C13—H13	0.9300
C6—C7	1.373 (3)	C14—C15	1.346 (4)
C6—C11	1.422 (3)	C14—H14	0.9300
C7—C8	1.396 (4)	C15—H15	0.9300
			
C5—S1—C2	91.16 (14)	C9—C8—H8	119.3
C1—N1—C6	119.0 (2)	C7—C8—H8	119.3
N1—C1—C2	123.0 (2)	C8—C9—C10	120.7 (3)
N1—C1—H1	118.5	C8—C9—H9	119.7
C2—C1—H1	118.5	C10—C9—H9	119.7
C3—C2—C1	127.8 (2)	C9—C10—C15	122.1 (2)
C3—C2—S1	110.6 (2)	C9—C10—C11	118.8 (2)
C1—C2—S1	121.4 (2)	C15—C10—C11	119.0 (2)
C2—C3—C4	113.2 (3)	C12—C11—C6	122.9 (2)
C2—C3—H3	123.4	C12—C11—C10	118.1 (2)
C4—C3—H3	123.4	C6—C11—C10	119.0 (2)
C5—C4—C3	112.1 (3)	C13—C12—C11	120.7 (3)
C5—C4—H4	124.0	C13—C12—H12	119.6
C3—C4—H4	124.0	C11—C12—H12	119.6
C4—C5—S1	112.9 (2)	C12—C13—C14	120.3 (3)
C4—C5—H5	123.5	C12—C13—H13	119.9
S1—C5—H5	123.5	C14—C13—H13	119.9
C7—C6—N1	123.1 (2)	C15—C14—C13	120.9 (3)
C7—C6—C11	119.8 (2)	C15—C14—H14	119.6
N1—C6—C11	117.2 (2)	C13—C14—H14	119.6
C6—C7—C8	120.2 (3)	C14—C15—C10	121.0 (3)
C6—C7—H7	119.9	C14—C15—H15	119.5
C8—C7—H7	119.9	C10—C15—H15	119.5
C9—C8—C7	121.3 (3)		
			
C6—N1—C1—C2	−178.1 (2)	C8—C9—C10—C11	−0.5 (4)
N1—C1—C2—C3	−178.9 (2)	C7—C6—C11—C12	−177.1 (2)
N1—C1—C2—S1	6.2 (3)	N1—C6—C11—C12	1.6 (3)
C5—S1—C2—C3	0.9 (2)	C7—C6—C11—C10	5.0 (3)
C5—S1—C2—C1	176.7 (2)	N1—C6—C11—C10	−176.3 (2)
C1—C2—C3—C4	−176.7 (2)	C9—C10—C11—C12	179.4 (2)
S1—C2—C3—C4	−1.3 (3)	C15—C10—C11—C12	0.6 (3)
C2—C3—C4—C5	1.0 (3)	C9—C10—C11—C6	−2.6 (3)
C3—C4—C5—S1	−0.3 (3)	C15—C10—C11—C6	178.6 (2)
C2—S1—C5—C4	−0.4 (2)	C6—C11—C12—C13	−179.9 (2)
C1—N1—C6—C7	−36.6 (3)	C10—C11—C12—C13	−2.0 (4)
C1—N1—C6—C11	144.7 (2)	C11—C12—C13—C14	2.0 (4)
N1—C6—C7—C8	176.9 (2)	C12—C13—C14—C15	−0.5 (4)
C11—C6—C7—C8	−4.4 (4)	C13—C14—C15—C10	−0.9 (4)
C6—C7—C8—C9	1.4 (4)	C9—C10—C15—C14	−177.9 (3)
C7—C8—C9—C10	1.1 (5)	C11—C10—C15—C14	0.8 (4)
C8—C9—C10—C15	178.3 (3)		

### Hydrogen-bond geometry (Å, °)

**Table:**

*D*—H···*A*	*D*—H	H···*A*	*D*···*A*	*D*—H···*A*
C12—H12···N1	0.93	2.52	2.837 (4)	100
C13—H13···Cg1^i^	0.93	2.87	3.783 (3)	168

Symmetry codes: (i) −*x*, −*y*, *z*.
